# Structure of the Fc fragment of the NIST reference antibody RM8671

**DOI:** 10.1107/S2053230X18009834

**Published:** 2018-08-29

**Authors:** D. Travis Gallagher, Connor V. Galvin, Ioannis Karageorgos

**Affiliations:** a NIST/IBBR, 9600 Gudelsky Drive, Rockville, MD 20850, USA; b New College of Florida, Sarasota, FL 34243, USA

**Keywords:** antibodies, NIST reference antibody RM8671, conformation, crystal structure, Fc, human, immune, X-ray

## Abstract

As the link between antigen binding and immune activation, the antibody Fc region has received extensive structural study. In this report, the structure of the Fc fragment of the NIST IgG1 mAb (reference material 8671) is described at 2.1 Å resolution in space group *P*2_1_2_1_2_1_, with approximate unit-cell parameters *a* = 50, *b* = 80, *c* = 138 Å. Prior Fc structures with a wide variety of modifications are also surveyed, focusing on those in the same crystal form. To facilitate the analysis of conformations, a reference frame and a two-parameter metric are proposed, considering the CH2 domains as mobile with respect to a fixed dimeric CH3 core. Over several human Fc structures, a significant variation in Fc elbow conformations is observed, which may serve to facilitate the regulation of Fc effector signaling.

## Abstract 2

The structure of the Fc fragment of the NIST reference antibody RM8671 is described in an orthorhombic crystal form. The molecular conformation is compared with those of precedents using a CH3-based reference frame and the pronounced asymmetry is linked to packing interactions.

## 1. Introduction   

The Fc (fragment crystallizing) of the IgG antibody was named by Rodney Porter, who found that it crystallized unexpectedly in his refrigerator (Porter, 1959 ▸). Using rabbit proteins, he was among the first to analyze the isolated Fab and Fc fragments; this work paved the way for molecular immunology and led to his 1972 Nobel Prize, which was shared with Gerald Edelman. The antibody Fc translates pathogen recognition into immune activation, but the regulation of this crucial signal involves a delicate balance dependent on structural details (Caaveiro *et al.*, 2015 ▸). The binding of Fc to various types of FcR cellular receptors is crucial and is sensitive to variations in the glycan that is attached to the conserved Asn300 (the actual sequence number varies owing to differing CDR lengths, and many structures call this residue Asn297). Other key binding partners include C1q (Schneider & Zacharias, 2012 ▸), which initiates the complement reaction, and neonatal FcRn (Oganesyan *et al.*, 2014 ▸), which facilitates the transport of maternal antibodies into newborns.

The immune system functions by deploying a wide repertoire of specific molecular shapes, so that structural knowledge of antibodies has become a key resource in understanding immunity and in the engineering of immune-inspired molecular therapies (Kennedy *et al.*, 2018 ▸). The Protein Data Bank (Berman *et al.*, 2000 ▸) contains over 100 structures of human IgG Fc crystallized with variations in the glycan, in the protein sequence and in the crystallization conditions, and in various complexes (see Supplementary Table S1 for a summary of precedent Fc structures). Complexes with FcR are especially informative and include most of the known FcR subtypes. The set of uncomplexed human Fc structures includes one common recurring crystal form in space group *P*2_1_2_1_2_1_, with approximate unit-cell parameters *a* = 50, *b* = 80, *c* = 138 Å. This common crystal includes about 40 structures, but for wild-type sequences its resolution is limited to about 2 Å. The highest resolution structures in this crystal form are at about 1.7 Å and contain multiple mutations. The highest resolution Fc structures in general are complexes such as PDB entry 1l6x (1.65 Å) and stabilized mutants such as PDB entry 5jih (1.7 Å). Supplementary Table S1 gives information on 16 representative precedent Fc structures.

In order to support the development of antibody-based medicines, the National Institute of Standards and Technology (NIST) has released an extensively characterized IgG1 mAb, called reference material 8671 or NISTmAb. To provide a scientific description of this material and to facilitate its application, we report the crystal structure of its Fc fragment. This is now the highest resolution structure available with its specific sequence in the common orthorhombic form. We compare it with other high-resolution structures, emphasizing the variations in overall conformation, and develop a reference framework based on two geometric parameters to facilitate conformational analysis.

## 2. Materials and methods   

### 2.1. Macromolecule production   

The crystallized Fc was prepared from a starting antibody that is a humanized IgGk1 mAb produced recombinantly in HEK293F cells and is available as NIST reference material 8671 (Schiel *et al.*, 2015 ▸). The Fc fragment was prepared from NISTmAb by papain cleavage as described in Karageorgos *et al.* (2017 ▸). After cleavage, the digest was applied onto a 5 ml protein A agarose column (Pierce Nab column, Thermo Scientific, Waltham, Massachusetts, USA) that had been equilibrated with 25 ml PBS and was incubated at room temperature for 20 min with agitation to capture the Fc and potential undigested NISTmAb. Fc and undigested mAb were eluted using the elution buffer from the Pierce Fab Preparation kit (Model 44985, Thermo Scientific) and Fc was separated from undigested mAb using a 100 kDa cutoff Amicon Ultra centrifugal filter (Sigma–Aldrich, St Louis, Missouri, USA) followed by preparative gel-filtration (GF) chromatography (Supplementary Fig. S1). Prior to GF chromatography, the material, both nonreduced and BME-reduced, was analyzed by gel electrophoresis (Supplementary Fig. S2). After GF, the collected Fc peak was dialyzed into 20 m*M* l-histidine buffer pH 6.0 and was concentrated to 25 mg ml^−1^ for crystal screening. Macromolecule-production information is given in Table 1 ▸.

### 2.2. Crystallization, data collection and structure determination   

Initial screening by vapor diffusion in sitting drops at 24°C produced crystals in ten out of 96 conditions. The conditions that gave the largest crystals were refined and optimized to reservoir conditions consisting of 40 m*M* CaCl_2_, 17%(*w*/*v*) PEG 8000, 100 m*M* sodium HEPES pH 7.0. Crystal growth was highly sensitive to the calcium concentration. Strontium appeared to substitute for calcium, but subsequent analysis failed to reveal any bound strontium site, suggesting that the role of the divalent cation in crystal growth is transient. Subsequent analysis of crystallization conditions in 38 Fc structures of the same crystal form in the PDB (with various mutations and glycoforms *etc.*) revealed that over 90% had a pH of between 5.8 and 7.2 and used some form of polyethylene glycol, but they were ionically diverse and only two included divalent cations. A crystal of dimensions 300 × 100 × 50 µm was dunked for 2 s into 15%(*w*/*v*) glycerol and 85% reservoir solution and then cryocooled in liquid nitrogen and kept at −100°C for data collection. Diffraction data were collected to 2.1 Å resolution on beamline 19-ID of the Advanced Photon Source (APS) at Argonne National Laboratory using an ADSC Q315r detector. Data in space group No. 19 were processed using *HKL*-3000 (Minor *et al.*, 2006 ▸). The previously observed unit cell implied that the crystal was isomorphous to several precedent Fc structures. Refinement began with a model derived from the precedent structure with PDB code 3do3 (B. C. Braden, unpublished work), from which the glycans and hinge-proximal loops had been removed. Refinement used *REFMAC* in the *CCP*4 suite (Winn *et al.*, 2011 ▸) and proceded in stages, gradually adding the missing parts and waters. In addition to general structure validation by the PDB, the glycans were validated by *pdb-care* at glycosciences.de (Emsley *et al.*, 2015 ▸).

### 2.3. Structure analysis   

**Figure 1:**
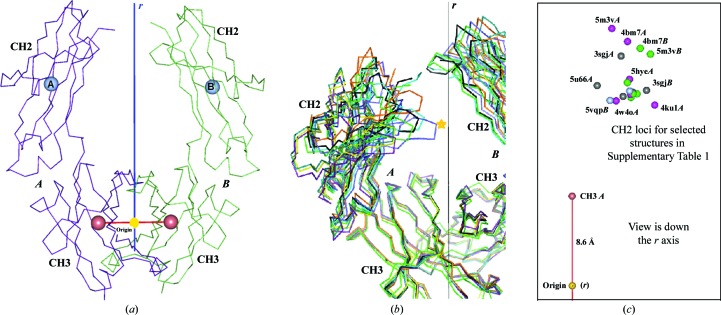
Geometric reference frame for Fc conformational analysis. (*a*) shows the least-squares dyad relating the tightly associated and structurally conserved CH3 domains. The centroids of the four domains are shown as spheres. Using this dyad as a vertical axis and the CH3 domain centroids to define the horizontal axes of a Cartesian frame, CH2 loci in different structures can be compared (after superposing their CH3 domains). (*b*) shows a superposition of seven Fc structures from the orthorhombic crystal form, showing that the CH2 domains have a wide range of positional variation. The star indicates the Asn residue to which the glycans attach. This point varies by up to 1.2 nm among the structures, suggesting that the glycans explore a large conformational space. (*c*) shows the same data using the proposed reference frame, and includes two additional Fc structures: the complexes with PDB codes 3sgj and 5u66.

*PyMOL* (DeLano, 2002 ▸) was used to generate molecular graphics as well as for structure alignment and r.m.s.d. calculations. *PISA* (Krissinel & Henrick, 2007 ▸) was used for the calculation of buried surface areas. Statistics for the deposited structure (PDB entry 5vgp) are given in Tables 2 ▸ and 3 ▸. A reference frame for conformational analysis was developed beginning from a Cartesian origin defined at the center of gravity of the CH3–CH3 dimer and a dyad passing vertically through this point (see Fig. 1 ▸ *a*). In some Fc structures this dyad is perfect (crystallographic), but in most it is only a local dyad and the two chains differ, giving a sample of conformational variation. The reference frame is then further defined by a horizontal plane through the centers of the two tightly associated CH3 domains. To avoid confusion with crystal axes, we label the reference axes *p*, *q* and *r*, with *r* as the vertical dyad. The centers of the two CH2 domains can then be plotted with respect to the dimeric CH3 core, and various structures with different elbow conformations can be superposed by their relatively rigid CH3 domains to observe the variation in their CH2 loci (see Fig. 1 ▸ *b*). Although in principle each CH2 centroid has three degrees of freedom, the elbow appears to function as a ball-and-socket joint (Teplyakov *et al.*, 2013 ▸) so that there are two main degrees of freedom, and the small vertical variation can be neglected. Using this system, Fig. 1 ▸(*c*) shows that a sampling of Fc structures, within the space group and beyond, shows a range of different conformations.

## 3. Results and discussion   

### 3.1. Structure of NISTmAb Fc and allotypic variation   

**Figure 2:**
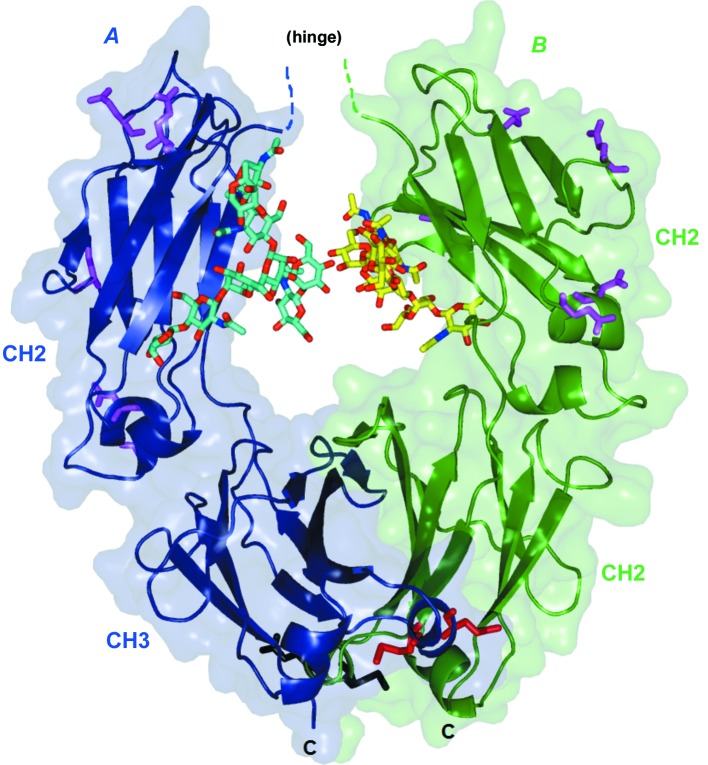
Overall arrangement of the Fc structure, with subunit *A* colored blue and subunit *B* in green. The N-terminal location of the hinge is at the top center and the C-termini are shown at the bottom. Each chain comprises two domains labeled CH2 and CH3. Glycans are shown centrally as bright sticks. Each subunit has seven sites of commonly observed allotypic variation, which are also shown as sticks (see §[Sec sec3]3). All figures were prepared using *PyMOL* (DeLano, 2002 ▸).

**Figure 3:**
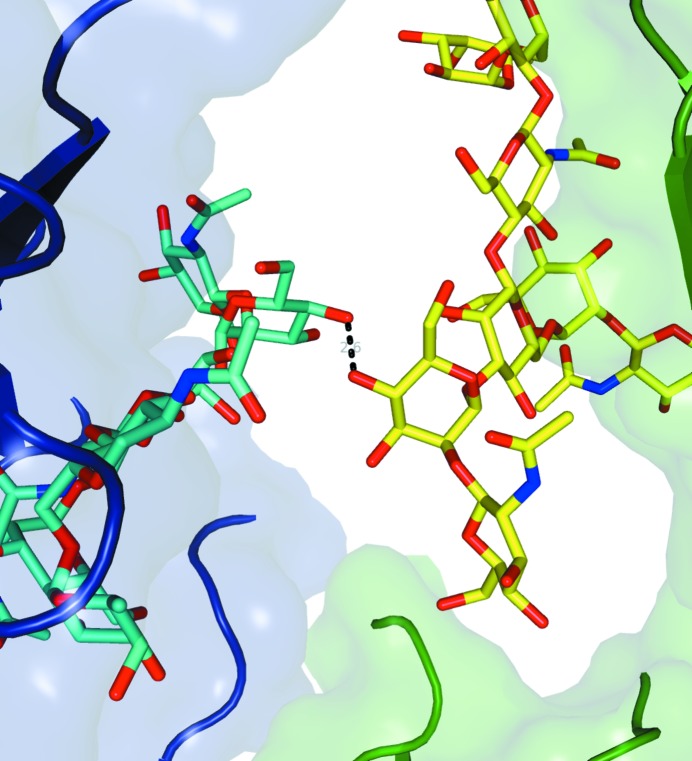
Close-up of the hydrogen bond at the central dyad between the two glycans.

The deposited structure includes all protein atoms from the hinge double glycine (residues 239 and 240) to Ser447 in both chains (Fig. 2 ▸). The hinge residues 228–238 are disordered. Although excluded from the refined structure, weak electron density was observed for the entire hinge, including both disulfides (data not shown); the space between adjacent molecules appears to be just right to accommodate the hinge, and this may help to explain the common occurrence of this crystal form among many variations in the Fc that preserve the IgG1 hinge. The crystals gave significantly better diffraction from the *A* chain relative to the *B* chain (the mean isotropic *B* values are 47 and 66 Å^2^, respectively). The glycans include all atoms consistent with the biophysically characterized principal glycoform G1F/G0F (*i.e.* the *A*-chain glycan has nine sugar groups including one fucose and one terminal galactose, while the *B* chain has only eight, lacking galactose). The two glycans interact through one hydrogen bond between mannose hydroxyls across the molecular dyad (Fig. 3 ▸). Other glycan interactions include several weak hydrogen bonds to the protein.

Most human Fcs with known crystal structures belong to one of three allotypes, correponding to polymorphisms that appear as sequence variations at seven specific sites, as shown in Fig. 2 ▸. In the structure with PDB code 5vgp these sites are Glu275, Glu286, Glu297, Asp315, Asn318, Glu359 and Met361. The first five of these are in CH2 and vary as a group, appearing as E275Q, E286Q, E297Q, D315N and N318D in the precedent structure PDB entry 1h3t and a few others. The last two of the seven variable sites are in CH3, and occur as E359D and M361L in PDB entries 4w4n, 5jii and several others. Allotypic variation in human populations is useful for identity testing but has unknown functional implications, and for therapeutic antibodies creates an unknown risk of immuno­genicity (Vidarsson *et al.*, 2014 ▸). Most of these sites are on the surface and most are involved (within 4 Å) in crystal-packing interactions so that they may also influence crystallization and resolution.

### 3.2. Symmetry and packing   

**Figure 4:**
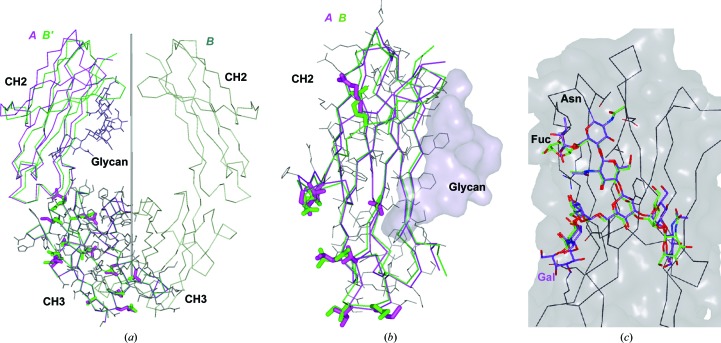
Superpositions showing the similarity of the two subunits in the structure with PDB code 5vgp. In (*a*) the alignment is based on the CH3 domains, which share a near-perfect dyad (the r.m.s.d. over 103 C^α^ atoms is 0.27 Å). The *A* chain is in magenta and the rotated *B* chain is in green. The CH2 domains show a misalignment of several angstroms since they do not maintain the CH3 dyad. The 12 side chains in CH3 with distinct rotamers in the two subunits (excluding fully solvated charged side chains) are shown as sticks. A single set of thin lines is used for CH3 side chains that agree. The glycan in this picture is for general location only. In (*b*) the two CH2 domains are superposed (the r.m.s.d. over 107 C^α^ atoms is 0.95 Å), showing that the loops (at top and bottom) deviate by about an angstrom. The nine side chains that have different rotamers in the two subunits are shown as sticks. In (*c*) the two glycans are superposed. The only galactose residue in the structure is at the lower left, where the *A*-chain glycan (magenta) has one additional saccharide unit. The fucose moieties at the upper left show distinct conformations, as do the glucosamines at the lower right.

Although the Fc is chemically symmetric or very nearly so, most Fc crystal structures are asymmetric. In the common orthorhombic crystal form in the present study, the asymmetric unit contains a complete Fc molecule and the two chains differ (Fig. 4 ▸). The differences are greatest in the CH2 domains, and in most structures one subunit is better ordered (with lower temperature factors) than the other. The better ordered (lower average *B*) subunit is generally the one that corresponds to the *A* chain in PDB entry 5vgp, which is the one that is further from the CH3-based molecular dyad (Fig. 4 ▸ *a*). This is consistent with the fact that the *A*-to-*A* crystal packing contact is significantly larger than the *B*-to-*B* packing contact (Supplementary Fig. S5).

The crystal organization builds on the dominant contact shown in Supplementary Fig. S3, which is unique in that it has nearly perfect twofold symmetry despite being between the *A* and *B* chains so that it is only pseudosymmetric. The crystal symmetry is pseudo-*C*222_1_. If we imagine rotating the Fc by about 11° so that its molecular dyad (arrow in Supplementary Fig. S4) aligns with the *z* axis, so that the contact in this figure becomes symmetric and the contacts in Fig. S5 become identical, then all of the pseudodyads would become dyads and the crystal would become *C*222_1_ with a single chain in the asymmetric unit. Presumably the actual packing is energetically preferred so that the molecule tends to crystallize in the observed lower symmetry. However, there are several reported Fc structures with the corresponding *C*222_1_ symmetry, for example PDB entries 3c2s, 5vh5 and 5u66.

The Fc dyad is the symmetry axis of the entire antibody, although the extreme flexibility of the hinge region means that the Fabs normally adopt unique and asymmetric conformations. The CH3–CH3 dimer forms the core of the dyad, with its central interface burying 1122 Å^2^ in PDB entry 5vgp. Since the CH2 domains and glycans do not appear to interact much (across the dyad), the only other tether holding the antibody together is the hinge disulfides. The Fc elbow between the CH2 and CH3 domains, spanned by residues 343–345 and burying about 40 Å^2^, has some flexibility, which enables the CH2 domains to adopt somewhat varied conformations (Fig. 1 ▸). Systematic analysis of this conformation variation may enable a more complete description of the energy landscape of Fc flexibility and the implications for effector binding. The proposed geometric framework applies regardless of crystal form; it does not even require a crystal. Since antibody effects follow the binding of monomeric FcR to both subunits of Fc, the intersubunit geometric relationship is likely to be important in signaling. The reference frame enables systematic correlation of Fc modifications (including glycoforms) with variations in the separation of the subunits and other intersubunit shifts that are likely to affect FcR binding.

## 4. Conclusion   

The structure of the Fc fragment from the NIST reference antibody RM8671 has been reported at a resolution of 2.1 Å (Fig. 2 ▸), along with a brief survey of precedent Fc structures and their conformational variation.

How antigen binding leads to multiple finely regulated effector functions is a central question in immunology. Homeostasis against an ever-changing barrage of microbial and other challenges requires that signaling interactions be information-rich and not simply on/off. The intensity of an immune response is an analog output, a matter of degree, suggesting that Fc signaling may involve regulation through some form of conformation-dependent code. This appears to apply to the large conformational variation of the hinge, where IgG subtypes have evolved various lengths and flexibilities, and hinge conformation affects the geometry of multivalent pathogen binding. Variation in the Fc elbows is also likely to be a factor in signaling, as it affects the separation of the CH2 domains, which would in turn affect the accessibility of the glycans to FcR binding.

## Supplementary Material

PDB reference: Fc fragment of human IgG1 antibody from NISTmAb, 5vgp

Supplementary Figures and Table.. DOI: 10.1107/S2053230X18009834/rl5165sup1.pdf

## Figures and Tables

**Table 1:** Source and sequence

Source organism	*Homo sapiens*
Expression host	HEK293F cells
Complete amino-acid sequence of the construct produced	TCPPCPAPELLGGPSVFLFPPKPKDTLMISRTPEVTCVVVDVSHEDPEVKFNWYVDGVEVHNAKTKPREEQYNSTYRVVSVLTVLHQDWLNGKEYKCKVSNKALPAPIEKTISKAKGQPREPQVYTLPPSREEMTKNQVSLTCLVKGFYPSDIAVEWESNGQPENNYKTTPPVLDSDGSFFLYSKLTVDKSRWQQGNVFSCSVMHEALHNHYTQKSLSLSPGK

**Table 2:** Data collection and processing

Diffraction source	Beamline 19-ID, APS
Wavelength (Å)	0.97934
Temperature (K)	100
Detector	PILATUS 6M
Crystal-to-detector distance (mm)	290
Rotation range per image (°)	0.8
Total rotation range (°)	180
Exposure time per image (s)	2
Space group	*P*2_1_2_1_2_1_
*a*, *b*, *c* (Å)	49.91, 79.96, 138.35
α, β, γ (°)	90, 90, 90
Resolution range (Å)	30.0–2.1 (2.17–2.13)
Total No. of reflections	207234
No. of unique reflections	30746
Completeness (%)	94.4 (71.0)
Multiplicity	6.7 (3.3)
〈*I*/σ(*I*)〉	7.2 (2.6)
*R*_r.i.m._ †	0.106
Overall *B* factor from Wilson plot (Å^2^)	40.032

† Estimated *R*_r.i.m._ = *R*_merge_[*N*/(*N* − 1)]^1/2^, where *N* is the data multiplicity.

**Table 3:** Structure refinement

Resolution range (Å)	12.00–2.116 (2.169–2.116)
Completeness (%)	94.2
σ Cutoff	0.0
No. of reflections, working set	29080 (1759)
No. of reflections, test set	1477 (97)
Final *R*_cryst_	0.201 (0.258)
Final *R*_free_	0.245 (0.313)
No. of non-H atoms	
Protein	3338
Ligand	209
Water	218
Total	3765
R.m.s. deviations	
Bonds (Å)	0.013
Angles (°)	1.656
Average *B* factors (Å^2^)	
Protein	56.3
Ligand	68.5
Water	50.5
Ramachandran plot	
Favored regions (%)	98.6
Additionally allowed (%)	1.2
