# Supplementary material for: Structure of the Fc fragment of the NIST reference antibody RM8671
Source: Acta Crystallogr F Struct Biol Commun. 2018 Aug 29;74(Pt 9):524–9. doi: 10.1107/S2053230X18009834 (PMC6130425; doi:10.1107/S2053230X18009834)
Supplement: Supplementary file 1 [file f-74-00524-sup1.pdf]

STRUCTURAL BIOLOGY
COMMUNICATIONS

Volume 74 (2018)

Supporting information for article:

Structure of the Fc fragment from the NIST reference antibody RM8671

Travis Gallagher, Connor V. Galvin and Ioannis Karageorgos

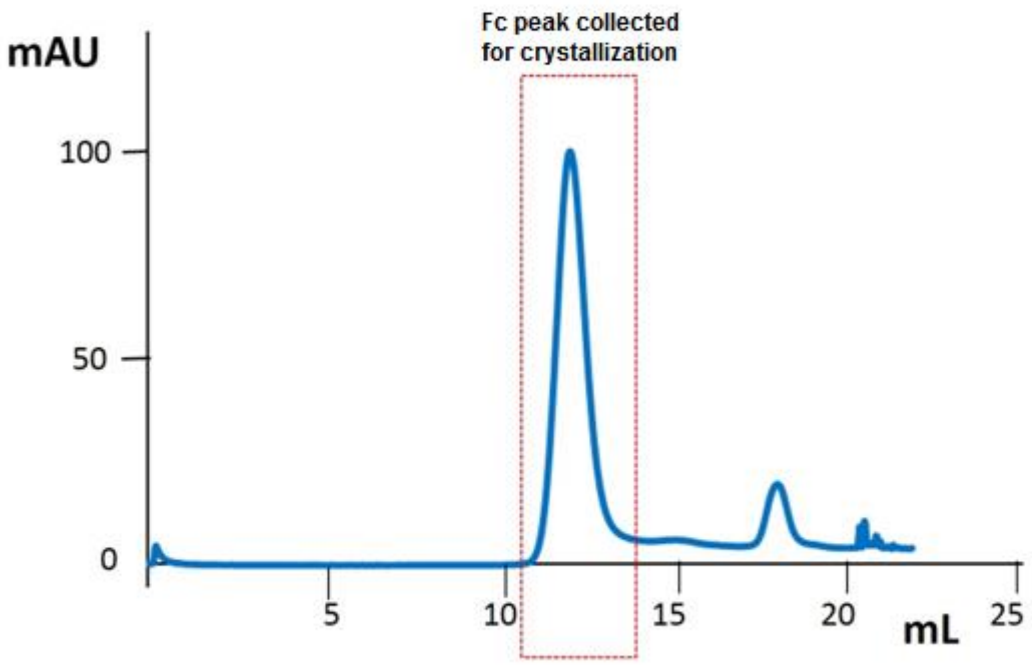

Figure S1 Elution profile from preparative gel filtration chromatography in PBS buffer. Injected sample of volume 100 uL was the filtrate from centrifugal filtration.

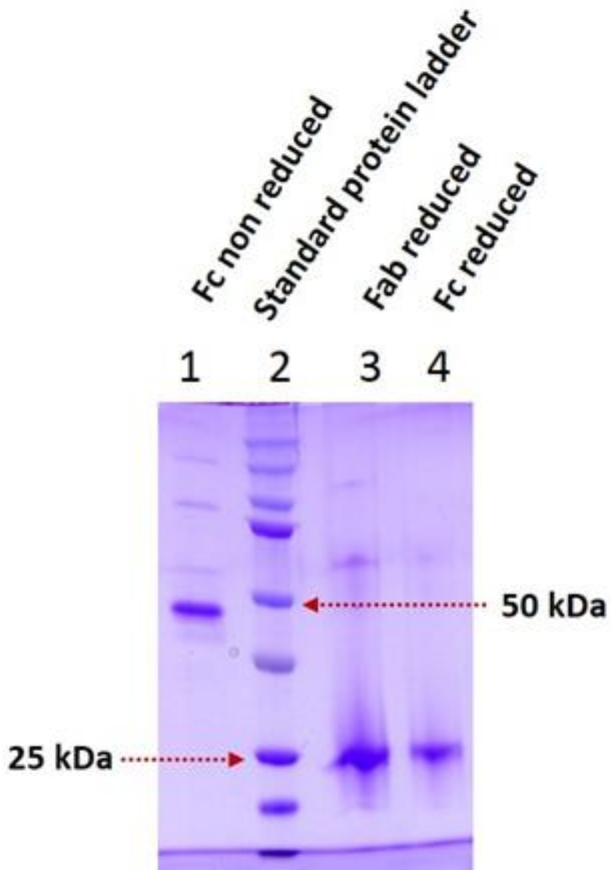

Figure S2 Polyacrylamide gel electrophoresis showing the nonreduced and reducd sample before GF chromatography. There is still a small amount of uncleaved mAb in the sample at this stage. Reduction by DTT converts the Fc from a 50 kDa band to a 25 kDa band, consistent with an intact hinge.

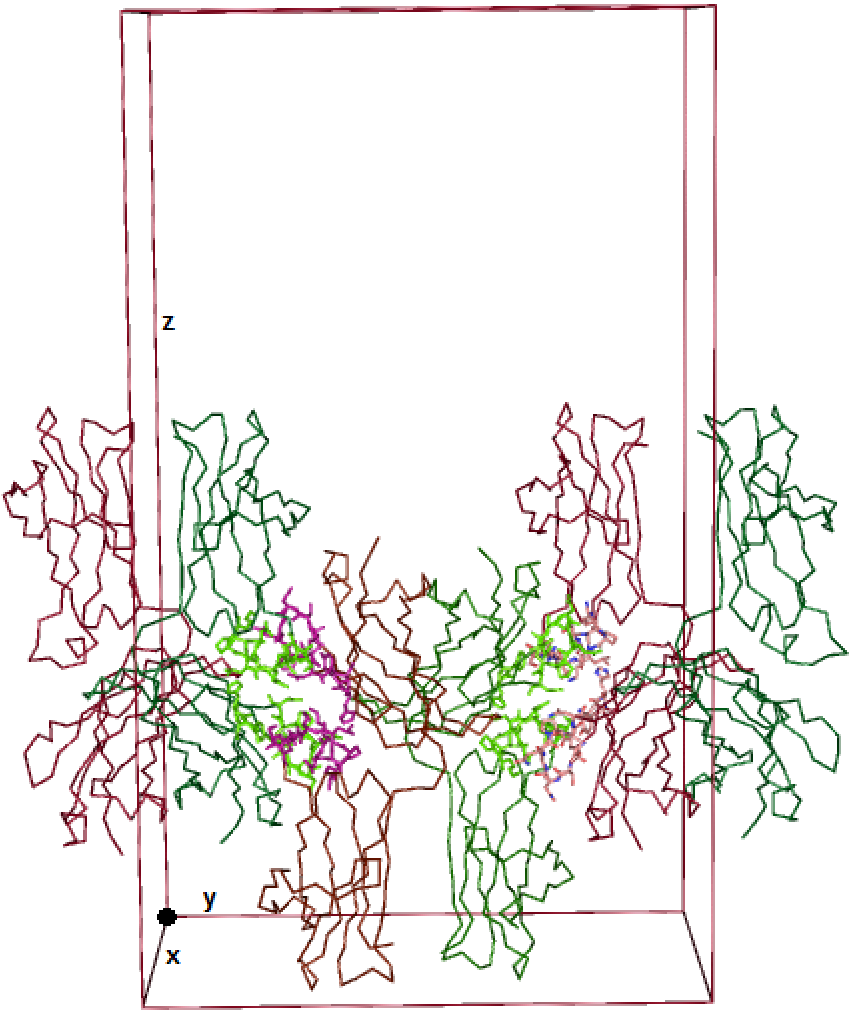

Figure S3 Crystal packing. This orthorhombic packing is shared by about 45 Fc structures. Figure S3 shows three Fc molecules in a chain connected by two instances of the largest contact (area 5.93 nm^2), which forms chains along the y direction. The contact occurs in the elbow region between the CH2 and CH3 domains and uses the same surface region where the biologically important binding to FcRn occurs. The contact relates A-chains to B-chains by a pseudodyad in the x direction that is locally almost perfect. Adjacent molecules are also related by a crystallographic screw along y.

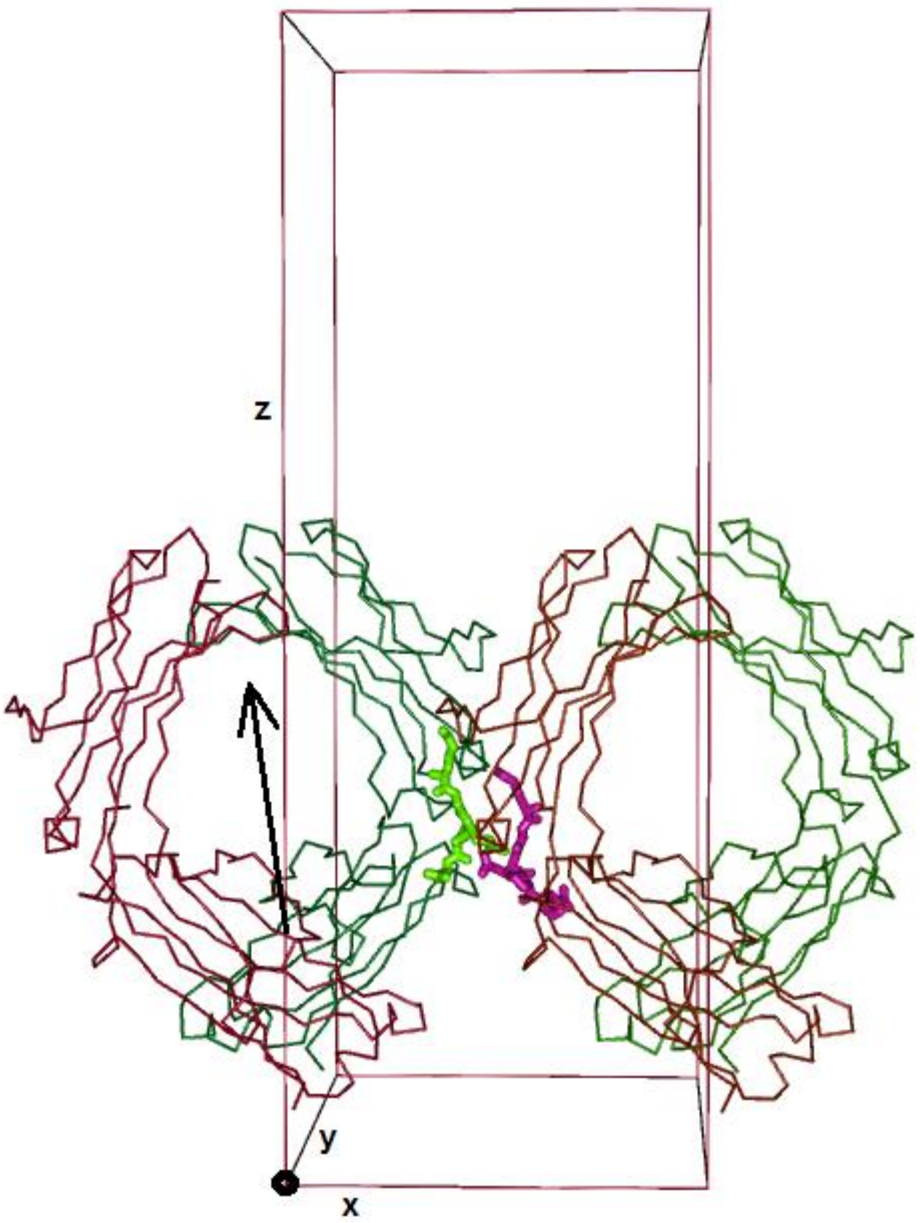

Figure S4 Two molecules connected by the second largest contact (area 3.20 nm^2), which forms chains along the x direction. This contact uses the elbow residues 343-345. Here molecules are related by pure translation, so that the crystal's **a** axis of 5.0 nm corresponds to a molecular diameter, and explaining why this cell dimension varies only slightly among different structures in this crystal form. Adjacent molecules are also related by a pseudodyad along the z direction, but it is distorted by a vertical shift of about 0.5 nm (green sticks mirror the red sticks but are above), and making the contact somewhat different for the two chains. Correspondingly, the pseudodyad thru the Fc molecule (arrow) deviates from the z direction by about 11˚. Those two contacts (in Figures S3 and S4) produce sheets of molecules in the xy plane, with the B chains projecting farther out of the plane than the A chains.

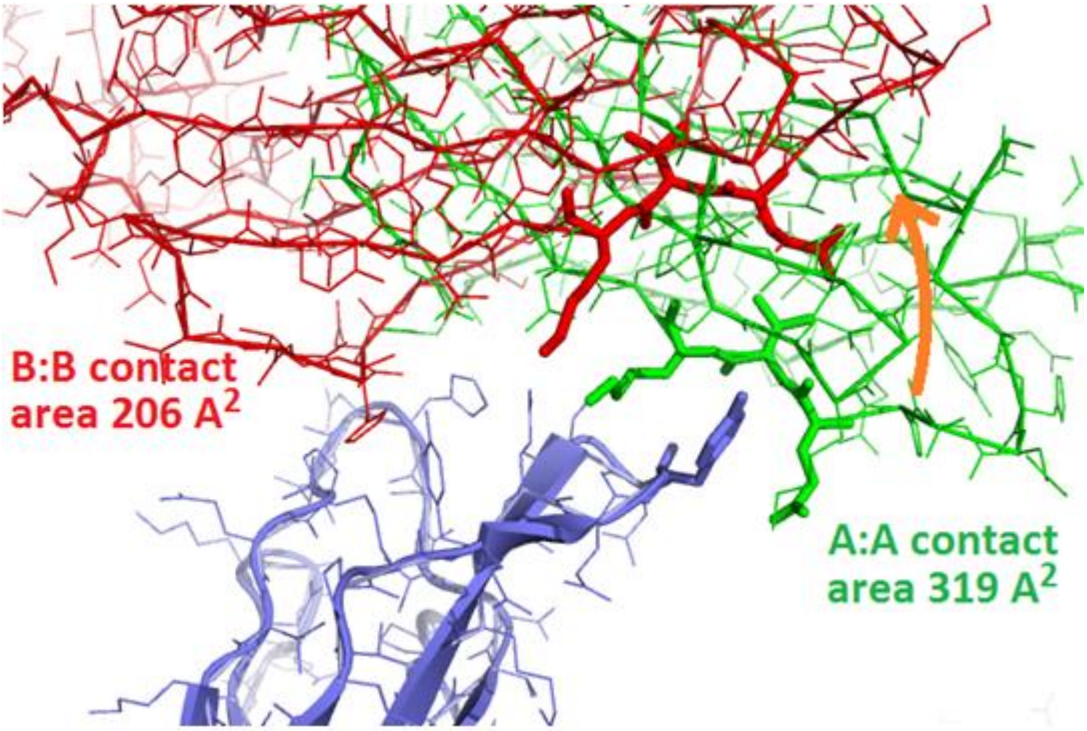

Figure S5 The two remaining contacts, which involve the CH2 tips (loops that are near in space to the Fc N-terminus) and serve to propagate the lattice in the z direction. These last two contacts are similar (both correspond to screw axes along the x direction) but they occur in an A-to-A form, which has area 3.19 nm^2, and a B-to-B form with area 2.06 nm^2.

Table S1 Representative Human IgG1 Fc Structures in the Protein Data Bank

Structures in the common orthorhombic crystal form (cell approx. 4.9 nm, 7.9 nm, 13.8 nm)

PDB ID	Resol. (nm)	Sequence/Allotype	Notes
5VGP	0.21	wild-type[a]	as reported in this paper
5JIH	0.17	10 mutations	affinity engineered
4BSV	0.18	2 mutations	heterodimer engineering
1H3T	0.24	315NwlD[b]	one of a glycoform variant series
4W4N	0.18	359DeL[c]	control for 4W4O (below)
5HYE	0.19	2 mutations	heterodimer engineering
4BM7	0.20	F241A	mutant affecting glycan dynamics
4KU1	0.19	359DeL	part of dynamics study
5M3V	0.20	21 mutations	heterodimer engineering

Structures in other crystal forms

4BYH	0.23	wild-type	sialylated glycan
5IW3	0.21	2 mutations	from anti-CD20 antibody

Structures of complexes

5U66	0.17	wild-type	Fc bound to Protein A helix
5U4Y	0.25	wild-type	Fc bound to Protein A
4W4O	0.18	359DeL	Fc bound to FcRI
3SGJ	0.22	359DeL	Fc bound to FcRIIIA
1I1A	0.28	6 mutations	Fc bound to FcRn

a. Wild-type here refers to the IgG1 Fc sequence corresponding to the Fc portion of Uniprot sequence Q6MZV7; see Results for a description of common allotypes.

b. This common allotype is differentiated by having NWLD in place of wild-type DWLN; see Results.

c. This common allotype is differentiated by having SRDEL in place of wild-type SREEM; see Results.
